# Evaluating the Precision of Automatic Segmentation of Teeth, Gingiva and Facial Landmarks for 2D Digital Smile Design Using Real-Time Instance Segmentation Network

**DOI:** 10.3390/jcm11030852

**Published:** 2022-02-06

**Authors:** Seulgi Lee, Jong-Eun Kim

**Affiliations:** 1 Department of Mechanical Engineering, Yonsei University, Yonsei-ro 50, Seodaemun-gu, Seoul 03722, Korea; sg.lee@yonsei.ac.kr; 2 Department of Prosthodontics, Yonsei University College of Dentistry, Yonsei-ro 50-1, Seodaemun-gu, Seoul 03772, Korea

**Keywords:** deep learning, digital smile design, digital dentistry, YOLACT++, detection, segmentation, 2D candid facial image

## Abstract

Digital smile design (DSD) technology, which takes pictures of patients’ faces together with anterior dentition and uses them for prosthesis design, has been recently introduced. However, the limitation of DSD is that it evaluates a patient with only one photograph taken in a still state, and the patient’s profile cannot be observed from various viewpoints. Therefore, this study aims to segment the patient’s anterior teeth, gingiva and facial landmarks using YOLACT++. We trained YOLACT++ on the annotated data of the teeth, lips and gingiva from the Flickr-Faces-HQ (FFHQ) data. We evaluated that the model trained by 2D candid facial images for the detection and segmentation of smile characteristics. The results show the possibility of an automated smile characteristic identification system for the automatic and accurate quantitative assessment of a patient’s smile.

## 1. Introduction

In dental prosthetic treatment process, especially the anterior dentition, it is important to restore the aesthetic smile through the production of prostheses as well as to improve the mastication function of the patient [1]. For this, it is important not only to acquire information about the shape, arrangement, and color of the patient’s existing teeth but also to understand whether anterior tooth alignment is in harmony with the major landmarks of the face and jawbone [2,3]. This process has been performed using the judgment of the clinician based on experience or using mechanical equipment, such as a facebow [4]. However, the experiences of clinicians vary greatly depending on their training in aesthetic judgment. In the case of the facebow, because it plays the role of transferring the relationship between the hinge axis and the maxilla to the articulator, it has the advantage of being able to set the occlusal plane information of the patient to the mechanical articulator [5,6]. However, it is difficult to accurately understand the aesthetic harmony with the patient’s face.

Recently, digital smile design (DSD) technology, which takes pictures of a patient’s face and anterior dentition together and uses them for prosthesis design, has been introduced [7,8]. Several related technologies and software have been developed and are extremely useful in clinical practice [7,8]. Before DSD technology, information confirmed, evaluated, and recorded by the clinician was delivered to the dental technician as a document, which is a limitation in information delivery as dental technicians had to produce prostheses that harmonize with the face of the patient without seeing the patient in person [9,10,11]. DSD has been evaluated as a useful technology that can overcome such limitations. Through this technology, dental technicians can obtain useful information for designing the prosthesis, such as the patient’s appearance, skin tone and whether the facial midline and the dental midline are parallel, without having to face the patient directly by using the photograph taken from the front. In addition, this technology assists in improving communication between dentists and dental technicians [12,13].

However, 2D DSD has a limitation in that it has to evaluate a patient with only one photograph taken in a still state, and the profile of the patient cannot be observed from various angles [13]. In addition, because the photographed smile is guided by a clinician, it might not be the natural smile of the patient, and it is difficult to photograph the ideal state [8,14]. A patient’s smile cannot be precisely reproduced; moreover, because the degree of laughter can be very diverse, it is not possible to grasp in detail the various teeth, gingival exposure and smile characteristics that a patient could have. Three-dimensional DSD technology using facial scan data can also obtain patient information from various directions; however, only one still image is recorded [7,8,15]. Although the analysis of the recorded photos is performed using software, it requires additional time and energy for analysis because it is a manual operation.

Recently, artificial intelligence (AI) has been actively applied in the health care field [16,17]. In particular, the convolutional neural network (CNN) shows excellent ability in detecting breast cancer, skin diseases and diabetic retinopathy through the learning of medical images or photos [18,19,20]. In the dental field, it is used to diagnose dental caries, and it is also applied to measure the alveolar bone loss due to periodontitis and classify implant systems [21,22]. Image segmentation technology is applied to tasks such as tooth numbering through tooth shape recognition, division of tooth destruction sites and apical lesions [23,24,25].

Up to now, there has been no study that performs the segmentation of tissues around the oral cavity such as teeth, gingiva and facial landmarks using deep learning based on facial photograph data and analyzes a patient’s smile. Therefore, the purpose of this study is to segment the teeth, gingiva and facial landmarks from the 2D candid facial image using a deep learning model for an automated smile characteristic identification system that analyzes the exposure of teeth and gingiva and the shape of the lips. The null hypothesis was that the average precision by individual class was over 0.8 at IoU thresholds of 0.5.

## 2. Materials and Methods

### 2.1. Dataset Description

Flickr-Faces-HQ (FFHQ), published online by NVIDIA Corporation, is a high-quality image dataset of human faces with high-quality PNG images with 1024 × 1024 resolution and considerable diversity in terms of age, ethnicity and image background [26]. The FFHQ dataset was used for the detection and segmentation of smiles and teeth in this study. The images used for the training contained general faces from various perspectives—excluding infants/children with primary teeth—and were resized to 550 × 550 resolution. In this study, it is essential to detect and segment individual teeth and gingiva; therefore, 80% of the dataset consisted of big smiling faces showing teeth and gingiva.

### 2.2. Dataset Annotation

Dataset annotation for deep learning was performed using an annotation tool Labelme for 40 objects:

- eye;
- eyebrow;
- nose;
- upper lip, lower lip;
- tragus;
- gingiva;
- buccal corridor;
- teeth (t11, t12, …, t18, t21, t22, …, t28, t31, …, t38, t41, …, t48).

**Figure 1:**
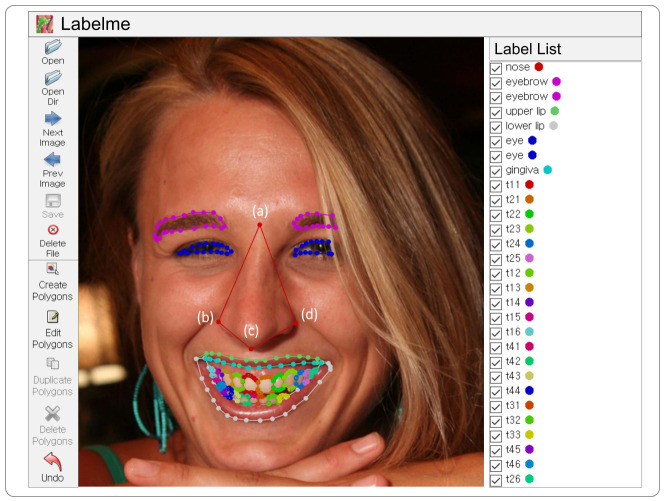
The example of annotations for the objects of smile information at a facial image using an annotation tool of Labelme: the nose annotation of polygons is performed using the four points as (**a**–**d**).

The type of annotation is polygons, and its boundaries must be clearly defined be-cause the annotated dataset is eventually used for the ground truth. Unlike other objects, the object of nose is difficult to define the boundary of the nose; hence, the nose annotation of polygons is performed using the four points shown in Figure 1. The 4 points consist of

(a) a point between eyebrows;
(b) a point located at the left ala of nose;
(c) a point falling to the pharynx;
(d) a point located at the right ala of nose.

Figure 1 shows the annotations for each object represented possibly in the facial image. The polygons annotation can be conducted using the button “Create Polygons” in Labelme and be finished by matching the starting point and ending point.

### 2.3. Deep Learning Model

The instance segmentation study, such as Mask R-CNN [27] and FCIS [28], relates to object detection, such as Fast R-CNN [29] and R-FCN [30]. However, such methods are focused on performance over speed. In this regard, YOLACT++ [31] aims to implement a one-stage instance segmentation model the same way as SSD [32] and YOLO [33]. However, the problem of dividing each class is significantly more complicated than the problem of object detection. Moreover, it is not easy to operate in real-time in the one-stage approach because a significant amount of post-processing is required after localization. To solve this problem, YOLACT++, an instance segmentation framework, gives up localization that requires extensive post-processing that allows it to operate in real-time [31]. The object segmentation step is performed as two parallel tasks: generating a non-local prototype mask for the entire image and predicting a set of linear combination coefficients per instance.

**Figure 2:**
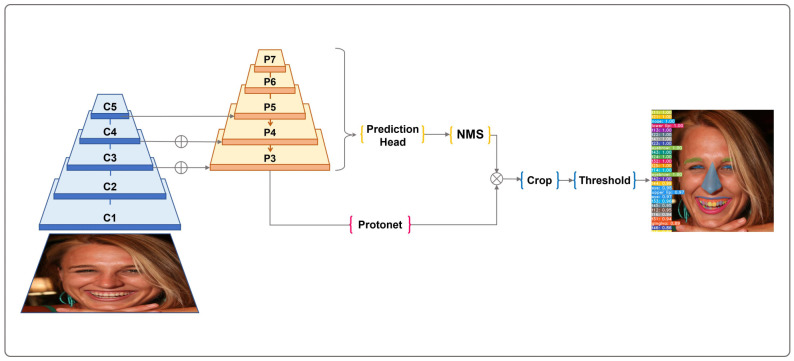
YOLACT++ architecture used for automated segmentation of teeth, gingiva and facial landmarks for digital smile design.

For the automated segmentation of teeth, gingiva and facial landmarks, we adopted YOLACT++ architecture based on RetinaNet [34] using ResNet-101 [35] with FPN [36]. YOLACT++ has introduced a novel fast non-maximal suppression (Fast NMS) and deformable convolutions into the encoder’s backbone network and a new mask rescoring branch [31]. The whole YOLACT++ architecture is depicted in Figure 2.

### 2.4. Model Training

The annotated FFHQ data were randomly split into three sets of 3300 for training, 1100 for validation and 1100 for testing. During training, the validation set was used to evaluate the performance of the model. After training, the test set was used to evaluate the performance of the model.

YOLACT++ used in the current research was implemented on Python3, Pytorch 10.0.1 and TorchVision. TorchVision is an open library for computer vision used with PyTorch. The model based on CNN is rarely trained from scratch because it requires a relatively large dataset. Therefore, the transfer learning technique was applied to the model trained with a batch size of eight on one GUP using ImageNet pretrained weights [37]. The model was trained with the stochastic gradient descent method [38,39,40] for 800,000 iterations starting at an initial learning rate of 0.001, with a momentum of 0.9 and weight decay of 0.0005, and all data augmentations used in the single-shot detector (SSD) [32] except up-side down and left/right flip were applied. The training process was conducted using a 3.0 GHz Intel Core i9-9980XE CPU, 62.5 GB RAM DDR4, GPU NVIDIA TITAN RTX 24 GB on an Ubuntu 20.04 operating system.

### 2.5. Statistical Analysis

In this simulation, in addition to the qualitative evaluation of visually comparing the segmentation results, the prediction performance of the model was evaluated quantitatively by calculating averaged precision (AP). AP is a popular metric for measuring object detection accuracy and can be obtained by integrating the precision–recall curve. There are three comprehensive evaluation indexes, namely precision (P), recall (R) and the intersection over union (IoU).

Precision measures how accurate the prediction is for the model and is defined as P = TP/(TP + FP), where TP and FP are true positive and false positive, respectively. Here, TP + FP is the total positive results predicted in the model. On the other hand, recall measures how well the model found all the positives and is derived as R = TP/(TP + FN), where FN denotes false negative and the denominator represents total ground truth. Unlike the classification model, the challenge of the detection model is to identify whether the results are true or not. Therefore, P and R can be resolved by introducing IoU, which measures how much the predicted boundary overlaps with the ground truth (the actual object boundary) and helps classify whether the prediction is true positive or false positive. In other words, if the IoU threshold is 0.5, the detection is correct when IoU ≥ 0.5.

## 3. Results

We report the results of automated instance segmentation of teeth, gingiva and facial landmarks using the standard metrics. The trained model is evaluated qualitatively and quantitatively using the test set.

**Figure 3:**
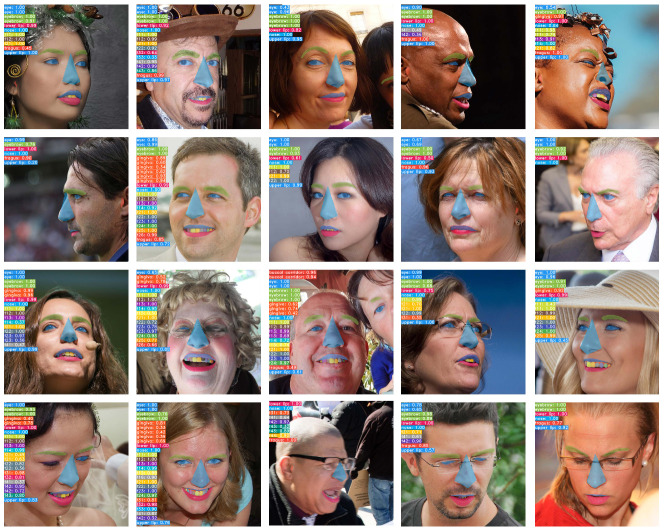
The overall segmentation results: the image of faces viewed from left, right and bottom to top are displayed in groups of rows in the aforementioned order.

The overall object segmentation results are illustrated with a confidence score greater than 0.3 in the face images shown as various viewpoints and numerous facial expressions in Figure 3. The qualitative results are output in the same color for each object, displaying the confidence score. Confidence scores are high for eyes, nose, lips and the smile-teeth showed when smiling. The smile-teeth defines the range of teeth of t13–t23.

### 3.1. Quantitative Segmentation Results

For an objective evaluation of our quantitative results, we first consider the APs according to the specific groups. The performance of the trained model was evaluated by grouping whole classes, eye, nose, lips and teeth as a superclass. The results for box and mask are represented in Table 1, where the subscript and superscript of AP denote the name of the superclass and the threshold of IoU, respectively. Here, no superscript of AP denotes the mean of the average precision and IoU of the range [0.5:0.05:095]. In the case of the mean IoU, ${\mathrm{AP}}_{\mathrm{all}}$ is around 0.3 for box and mask, and only ${\mathrm{AP}}_{\mathrm{nose}}$ is over 0.8. For an IoU threshold of 0.5, AP is over 0.9 as performance for eye, nose and lips, except teeth.

**Figure 4:**
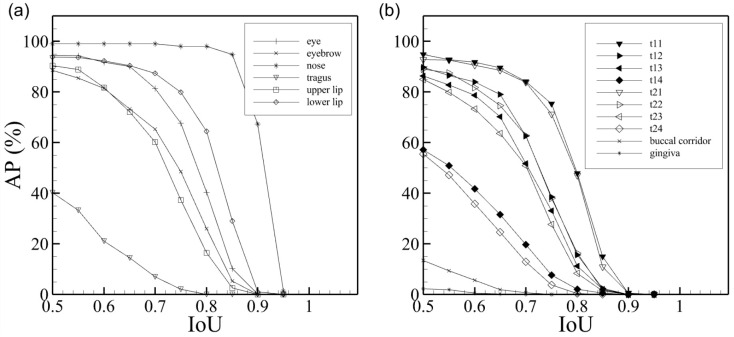
Average precision for smile characteristic identification mask at IoU threshold of 0.50–0.95: (**a**) AP of facial landmarks; (**b**) AP of intraoral structures.

We closely examine the AP for individual masks according to the IoU to evaluate the segmentation of teeth. The results AP with IoU for individual masks are depicted in Figure 4, wherein (a) shows AP for face information and (b) shows AP for smile-related information. When the IoU is 0.5, this trained model reached more than 80% accuracy for the eyes, nose, lips and smile-teeth (t13 to t23).

### 3.2. Qualitative Segmentation Results

**Figure 5:**
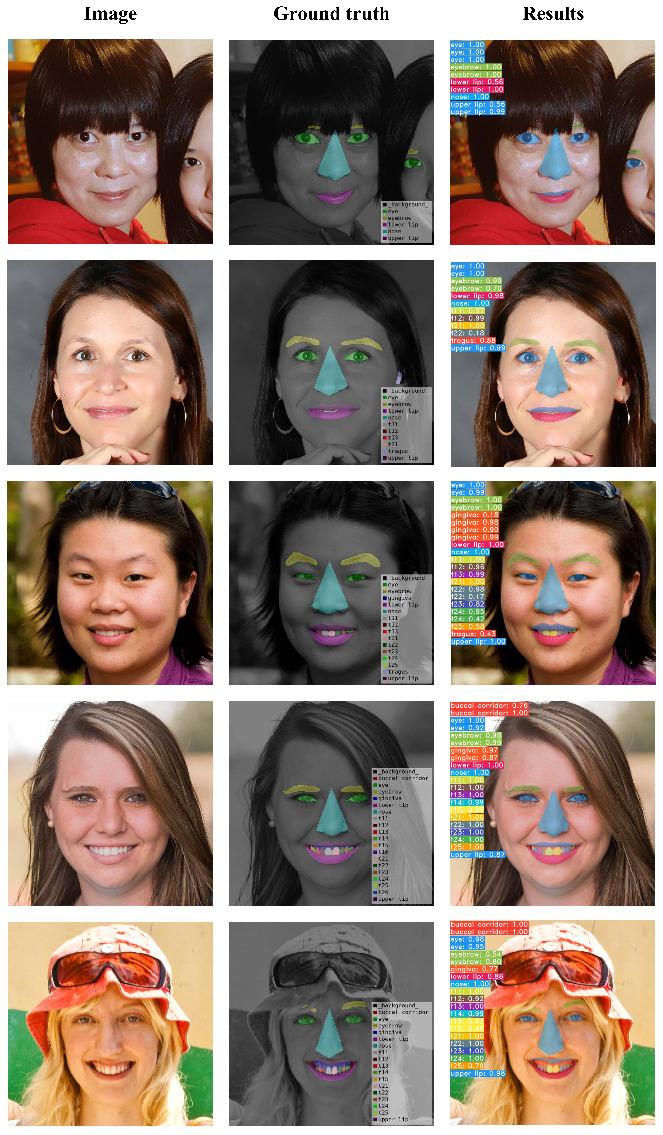
The comparison between the ground truth and segmentation results with the difference in smile: the smile grows as the image goes from top to bottom.

The qualitative segmentation results are illustrated in Figure 5 where the images by type of smile are arranged in columns. The results qualitatively show that the trained model achieved high detection accuracy. Moreover, gingiva and buccal corridor are detected well even though the AP is below 0.6 when IoU = 0.5.

## 4. Discussion

Recently, many researchers have been studying the use of deep learning in the dental field. In particular, there are a bunch of deep learning studies using a patient’s medical image or photos; however, studies with 2D images have not begun yet for DSD. The objective of this study was to segment teeth, gingiva and facial landmarks from a 2D candid image using deep learning. For the metric used in the Pascal VOC challenge [41], multi-class accuracy (${\mathrm{AP}}_{\mathrm{all}}^{50}$) is 0.472, but the ${\mathrm{AP}}^{50}$ for the smile characteristic identification, except for tragus, buccal corridor and gingiva, is over 0.8, which may have positively affected performance. Therefore, the null hypothesis was partially rejected.

The dataset used in this model consists of images of faces with different expressions from different angles. In Figure 3, the image of faces viewed from left, right and bottom to top are displayed in groups of rows in the aforementioned order. Therefore, it can be expected that, in addition to the 2D candid image, the detection according to the facial expression change in the interview video will be possible in this real-time instance segmentation network. The small and minute objects, such as the gingiva, tragus and teeth, were divided appropriately. The accuracy for each segmentation and the confidence score were high. Although the reliability score of the gingiva was lower than that of the other objects, the localization of the actual gingiva was relatively well detected.

The trained model was evaluated through the AP metrics represented in Table 1. ${\mathrm{AP}}_{\mathrm{all}}$ and ${\mathrm{AP}}_{\mathrm{all}}^{50}$ appear low at 0.229 and 0.341, respectively. For this simulation, there is no reference data that have the same classes, so if we compare the results of the MS COCO dataset [42] obtained by YOLACT++, our results fall behind by 0.117 for ${\mathrm{AP}}_{\mathrm{all}}$ and 0.066 for ${\mathrm{AP}}_{\mathrm{all}}^{50}$. This, however, does not mean the segmentation performance falls behind [31] because the training condition is different; 1475 images per class were used in the MS COCO dataset, while 82.5 images per class were used in our dataset. From Choi et al. [43], the accuracy of 80 images per class can yield about 0.85 (refer to null hypothesis). Therefore, the number in our dataset was adequately chosen.

${\mathrm{AP}}_{\mathrm{all}}$ is not the ideal measure of model performance. Therefore, AP was obtained by dividing the specific groups into the eyes, nose, lips and teeth, as listed in Table 1. ${\mathrm{AP}}_{\mathrm{nose}}$ shows the highest performance. In the case of nose segmentation, it was simplified and expressed as a rectangle using four points; because the labeling for each nose is unconditionally included in all photos, it contains a more significant amount of information than other objects; thus, it has good performance. However, although there are more eye annotations than the nose, ${\mathrm{AP}}_{\mathrm{eye}}$ is lower than ${\mathrm{AP}}_{\mathrm{nose}}$ because the eyes are not uniform in shape and vary—the states of half-opened eyes, closed eyes and the shape of the eyes are different. ${\mathrm{AP}}_{\mathrm{teeth}}$ appears relatively small. However, we can explain through Figure 3 our desired level of detection and segmentation.

For accurate performance analysis, we plotted the AP for each object according to the IoU threshold in Figure 4. Because true and false object detection are determined based on the IoU threshold, as the IoU threshold decreases, the true positive exists more. Therefore, it is essential to observe AP according to the IoU threshold. When the IoU threshold is 0.5, the model is well-trained for face information because APs, except for the tragus, are over 80%. When the IoU < 0.85, the AP for nose maintains a value of 90 or higher. The upper lip has a smaller AP than the lower lip because the change in the upper lip is more significant than the lower lip, depending on the expression. Object segmentation of face information, except for the tragus, show good performance. The tragus AP appears lower than that of other face objects because it is challenging to detect the tragus from the front; it is better detected from the side view. This can be confirmed qualitatively by the segmentation results in Figure 3 and Figure 5.

Likewise, the AP for smile information is represented in Figure 4b. We only show fourteeth, the smile-teeth, the buccal corridor and gingiva. The model performance for smile-teeth is more than 80% when the IoU threshold is 50. However, the APs for a tooth positioned far from the first teeth (t11, t21) are lower. This is related to the frequency of tooth exposure when smiling, and the performance is lower than other objects due to the lack of labeling. In other words, the further away from t11 and t21, the lower the AP is. In the case of the buccal corridor, the number of its annotations is inevitably smaller than the other objects and is more special for the smile; thus, the size of the training set is inevitably insufficient. Therefore, it shows a low AP compared to the other objects. Nevertheless, the model performed well in the buccal corridor and gingiva detection and segmentation, as illustrated in Figure 5.

Before the description of Figure 5, we discuss the results by Boiko [44], who performed Mask R-CNN using hyperspectral images to automatically segment enamel and attached gingiva. They achieved high-quality performance on enamel and gingiva; the AP for enamel is 0.99 at a IoU threshold of 0.77, and the AP for attached gingiva is 0.96 at a IoU threshold of 0.68. The number of classes was few, and trained hyperspectral images had clear information on enamel and gingiva. However, teeth and gingiva from 2D candid facial images are more complicated in our simulation condition. Therefore, our accuracy is low, but this study is a positive challenge.

Figure 5 shows the segmentation results obtained using the test set. In the column direction, the smile is gradually large, and in the row direction, raw data, ground truth and results are presented in order. The ground truth result in the first row shows that the person’s upper and lower lips on the right of the image are not annotated. However, our trained data detected the upper and lower lip on a half-visible face. Although the actual lips are correct, it is not a positive value for the ground truth; hence, it is one of the reasons for the low performance. The second row shows that teeth segmentation between the lips in the resulting image and the tragus segmentation was performed well. Even the tiny gingiva not annotated in the ground truth was segmented, as shown in the third row. The exposure of the gingiva increases, and thus, gingiva detection becomes more sophisticated. We annotated the buccal corridor for a smile analysis, and the model detected the buccal corridor well as per the results in the fourth and fifth rows. Therefore, such smile information obtained by deep learning can be used as a smile analysis parameter [7] in the future.

Recently, DSD technology, which has received remarkable attention from clinicians, facilitated communication during the dental prosthesis manufacturing process and contributed to improving the quality of the prostheses. However, because all processes were conducted manually, a significant amount of time was required for the work process; hence, it was not a convenient method. Using the results presented in this study, major landmarks on the patient’s face can be detected and segmented. Therefore, it is possible to easily grasp the characteristics of a patient’s smile using only a 2D candid facial image. For the segmented area, information such as area or width can be digitized, and the digitized result value and the characteristics of a smile can be classified.

However, this study also has limitations. Because this study is based on a 2D face photograph, it is very helpful in understanding the patient’s smile characteristics, but it is difficult to directly apply to the alignment of 3D dental arch data and the design process of modeling data based on it for the actual clinical procedure. For this purpose, if an automated system based on 3D data is developed, the scope of application can be further expanded. In this study, 2D candid images were analyzed. However, because video data can also be analyzed using the network used in this study, when conducting interviews with patients, the exposure of teeth or gingiva according to the smiles and expressions of various patients can be measured based on the interview images. It is possible to calculate and extract frames for various situations, such as the largest smile and the slightest smile, and it is possible to simply calculate the exposure frequency according to the characteristics of the smile. More meaningful data can be obtained because this information is obtained through an image rather than a single piece of a photograph. In the future, it is possible to relate the pronunciation of the patient in the image with the tooth exposure information, and it can lead to the development of applications such as analyzing the shape and wear of teeth. The ripple effect is expected to be significantly large.

## 5. Conclusions

We evaluated the method based on YOLACT++ trained by 2D facial images for detecting smile information including eye, eyebrow, nose, lips, gingiva, buccal corridor and smile-teeth. Even though APs for the gingiva and buccal corridor are relatively low, their predictions are not wrong in quality (Figure 5). Of course, more annotated gingiva and buccal corridor data for the training are needed for high-quality performance. Nevertheless, this is an excellent signal to analyze the exposure of gingiva related to smile information. Therefore, we can optimize the segmentation for smile information and propose an automated smile characteristic identification system for analyzing the characteristics of a patient’s smile in the future.

## Figures and Tables

**Table 1:** The specific group APs for box and mask: the superscript and subscript of AP denote IoU threshold and the name of a superclass (group), respectively.

Type	${\mathrm{AP}}_{\mathrm{all}}$ (${\mathrm{AP}}_{\mathrm{all}}^{50}$)	${\mathrm{AP}}_{\mathrm{eye}}$ (${\mathrm{AP}}_{\mathrm{eye}}^{50}$)	${\mathrm{AP}}_{\mathrm{nose}}$ (${\mathrm{AP}}_{\mathrm{nose}}^{50}$)	${\mathrm{AP}}_{\mathrm{lips}}$ (${\mathrm{AP}}_{\mathrm{lips}}^{50}$)	${\mathrm{AP}}_{\mathrm{teeth}}$ (${\mathrm{AP}}_{\mathrm{teeth}}^{50}$)
Box	0.341 (0.635)	0.621 (0.946)	0.879 (0.990)	0.645 (0.942)	0.303 (0.604)
Mask	0.229 (0.472)	0.570 (0.945)	0.855 (0.990)	0.541 (0.921)	0.175 (0.411)

## Data Availability

The data presented in this study are available on request from the corresponding author.
